# The utilisation of operant delayed matching and non-matching to position for probing cognitive flexibility and working memory in mouse models of Huntington's disease

**DOI:** 10.1016/j.jneumeth.2015.08.022

**Published:** 2016-05-30

**Authors:** Emma Yhnell, Stephen B. Dunnett, Simon P. Brooks

**Affiliations:** The Brain Repair Group, Cardiff University School of Biosciences, The Sir Martin Evans Building, Museum Avenue, Cardiff CF10 3AX, South Glamorgan, United Kingdom

**Keywords:** Huntington's disease, Operant tests, 9-Hole box, Reversal learning, Short-term memory, HD mice

## Abstract

**Background:**

Operant behavioural testing provides a highly sensitive and automated method of exploring the behavioural deficits seen in rodent models of neurodegenerative diseases, including Huntington's disease (HD). The delayed matching to position (DMTP) and delayed non-matching to position (DNMTP) tasks probe spatial learning and working memory and when applied serially they can be used to measure reversal learning, which has been shown to be an early symptom of executive dysfunction in HD.

**New method:**

The DMTP and DNMTP tasks were conducted in two configurations of operant apparatus; the conventional 9-hole operant apparatus, and a Skinner-like operant apparatus, to compare, contrast and optimise the DMTP and DNMTP operant protocols for use in mice. The optimised tasks were then tested in the Hdh^Q111^ mouse model of HD.

**Results:**

Optimisation of the operant apparatus demonstrated that the mice learned the DMTP and DNMTP tasks more rapidly and effectively in the Skinner-like apparatus configuration in comparison to the conventional 9-hole apparatus configuration. When tested in the Hdh^Q111^ mouse model of HD, the DMTP and DNMTP tasks revealed significant deficits in reversal learning.

**Comparison with existing method:**

We found that mice were capable of performing the DMTP and DNMTP tasks in both apparatus configurations, but in comparison to the 9-hole configuration, the Skinner-like configuration produced more efficient, robust and reliable results.

**Conclusions:**

The results presented here suggest that DMTP and DNMTP tasks, incorporating a reversal learning manipulation, are valid and robust methods for probing selected cognitive deficits in mouse models of neurodegenerative diseases.

## Highlights

• We compared delayed matching and non-matching to position (DMTP and DNMTP) tasks in two different operant apparatus, the 9-hole operant apparatus configuration and the Skinner-like operant apparatus configuration.
• We determined that the DMTP and DNMTP operant tasks produce more efficient, robust and reliable results in the Skinner-like operant apparatus configuration.
• We therefore used the Skinner-like operant apparatus configuration to test DMTP and DNMTP tasks in the Hdh^Q111^ mouse model of HD.
• We tested the DMTP and DNMTP tasks in the Hdh^Q111^ knock-in mouse model of HD which revealed significant deficits in task acquisition and reversal learning in comparison to wildtype animals.

## 1 Introduction

Since the discovery of the genetic cause of Huntington's disease (HD) (MacDonald et al., 1993) a wide range of genetically modified animal models of the disease have been created that demonstrate good construct and face validity to HD. By far the most widely used animal as a model of HD is the genetically modified mouse, due to the highly conserved genome in relation to the human genome and the comparative ease of genetic manipulation. Understanding the nature and severity of HD disease progression in these models is central to determining the suitability and predictive validity of these animals for therapeutic trials. There are now over 20 mouse models of HD (transgenic and knock-in) that have been reviewed extensively elsewhere (Hickey and Chesselet, 2002, Menalled and Chesselet, 2002). Whilst these mouse models demonstrate a range of behavioural abnormalities, there is still a lack of sensitive, reliable and robust behavioural tasks available to probe the specific cognitive deficits observed in HD.

The large number of mouse models of HD that are now available means that there is a need to continually develop novel behavioural tasks to better understand, validate and explore the behavioural symptoms that are demonstrated in HD mouse models. The use of the rat in many previous behavioural studies means that often behavioural tests for mice are modified from those traditionally conducted and developed for the rat (Brooks and Dunnett, 2009). The delayed matching to position (DMTP) and delayed non-matching to position (DNMTP) tasks are examples of such behavioural tests. The delayed matching tasks have been used extensively in a range of species including; monkeys (Mello, 1971, Bartus and Johnson, 1976, Hudzik and Wenger, 1993, Terry et al., 1993), pigeons (Blough, 1959, Ferster, 1960, Harnett et al., 1984, Urcuioli, 1985, Picker et al., 1987) and humans (Owen et al., 1995), often for testing neurological conditions such as Alzheimer's disease, Parkinson's disease and schizophrenia (Irle et al., 1987, Sahakian et al., 1988, Elliott et al., 1998). The DMTP and DNMTP tasks were used in rats to explore the effects of lesions and associated drug treatments (Dunnett, 1985, Dunnett et al., 1989). Since the original description in the rat, operant DMTP and DNMTP testing has been used in numerous other rat studies (Dunnett et al., 1988a, Bushnell, 1990, Cole et al., 1993, Carter et al., 1995, Stephens and Cole, 1996, Yamada et al., 2005). However, the use of DMTP and DNMTP tasks in mouse studies has been comparatively limited (Beracochea and Jaffard, 1995, Estapé and Steckler, 2001) and DMTP and DNMTP protocols have yet to be extensively investigated in HD mice. In HD, reversal learning deficits are a particular feature of both the human disease (Lawrence et al., 1998, Lawrence et al., 1999) and the HD mouse (Lione et al., 1999). Using the DMTP and DNMTP tasks in sequence and serially allows us to utilise a reversal learning shift in conjunction with a working memory probe in murine models of HD.

As an increasing number of mouse models of neurological diseases, including HD, become readily available, the DMTP and DNMTP tasks (and their subsequent reversals) need to be developed and optimised for use in mice. Although maze variations of the DMTP and DNMTP tasks have been previously performed using a T-maze experimental design (Gibbs, 2002, Johnson et al., 2002, Fitz et al., 2008), this type of behavioural testing is time consuming and provides minimal amounts of data, relative to the automated mass-trials produced by operant procedures, and it is susceptible to experimenter bias. Therefore, conducting DMTP and DNMTP tasks using operant behavioural testing methods presents a fully automated, sensitive and flexible way of measuring task performance. Therefore, the aims of this study were to compare and contrast DMTP and DNMTP protocols in two different configurations of the ‘9-hole box’ operant apparatus: a conventional configuration with central and lateralised response holes within a 9-hole array and with the reward hopper located in the opposite wall, and, a ‘Skinner like’ box configuration in which just two response holes were located one on either side of the central reward hopper. We then used the more efficient protocol to test the DMTP and DNMTP tasks in the Hdh^Q111^ mouse model of HD.

## 2 Materials and methods

### 2.1 Animals

Animals were maintained on a 12 h light/dark circadian schedule (0600 h lights on; 1800 h lights off), in a temperature controlled environment (21 °C ± 2 °C). Animals were housed in pairs, although sometimes had to be separated and singly housed to prevent fighting. Operant testing occurred during the light phase between 0800 h and 1100 h, five days a week. All animals were water restricted and habituated to strawberry milk (Yazoo^®^, Campina Ltd, Horsham, UK) in their home cages one week prior to operant testing. During operant testing, animals were maintained on a water restriction schedule of 3 h water, available daily from 1200 h to 1500 h in their home cages.

The C57BL/6J animals used in the comparison of the DMTP and DNMTP tasks in differing operant apparatus were obtained from Harlan (Bicester, Oxfordshire, UK) at 8 weeks of age. 14 C57BL/6J male animals were used in testing of the DMTP and DNMTP task in the conventional 9-hole apparatus and 15 C57BL/6J male animals were used in the testing of the DMTP and DNMTP task in the Skinner-like apparatus. Hdh^Q111^ animals were originally obtained from Jax^®^ (Jackson Laboratories, Bar Habor, Maine) and bred in-house on a C57BL/6J background. For the testing of the DMTP and DNMTP tasks in a mouse model of HD, a total of 21 littermate animals were used, 12 Hdh^Q111/+^ (6 were female and 6 were male) and 9 wildtype (5 were female and 4 were male). Animals were weaned at 3–4 weeks of age and tail tipped for genotyping (Laragen Inc., Culver City, CA, USA). CAG repeat length in Hdh^Q111/+^ animals ranged from 134 to 145 repeats, with an average repeat length of 140 repeats. Hdh^Q111/+^ animals began operant testing at 8 months of age. All testing was in accordance with the European Directive 2010/63/EU and the UK Animal and Scientific Procedures Act (ASPA) of 1986 and subject to local ethical review.

### 2.2 Apparatus

**Fig. 1:**
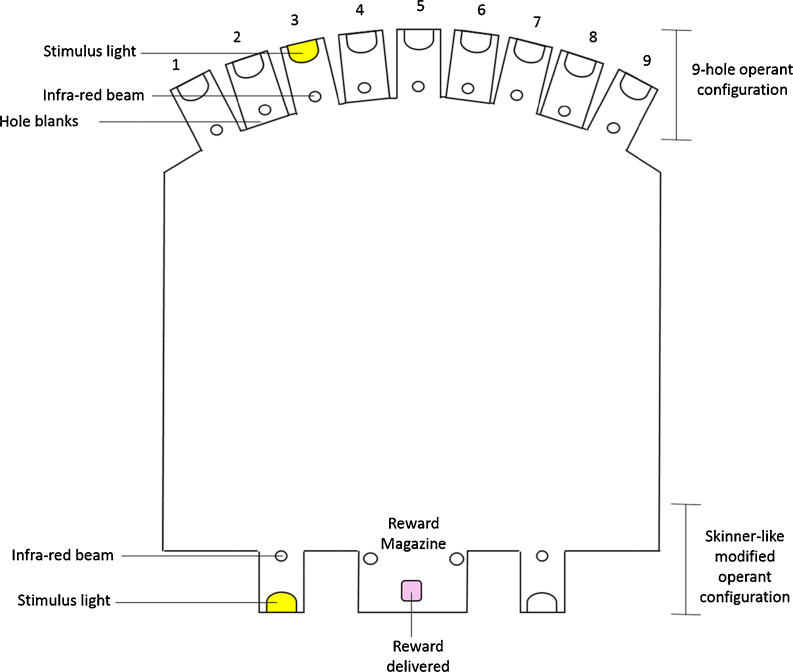
Schematic representation of the 9-hole and Skinner-like operant configurations. The 9-hole operant apparatus (top) included 9 response holes, (11 mm in diameter, placed 2 mm apart and 15 mm above floor level) although only 5 of these were used, holes 2, 4, 6,and 8 were covered using blanks. The reward magazine was located opposite the 9-hole array. The Skinner-like operant configuration (bottom) included 2 response holes (11 mm in diameter, 20 mm laterally from the reward magazine and 15 mm above floor level) located either side of the reward magazine. For both operant configurations the same reward magazine was used.

The operant apparatus used here comprised two separate operant configurations, a 9-hole operant apparatus configuration and a Skinner-like operant apparatus configuration, as shown in Fig. 1.

#### 2.2.1 Conventional 9-hole operant box configuration

Sixteen 9-hole operant boxes (Campden Instruments, Loughborough, UK), measuring 14 cm × 13.5 cm × 13.5 cm, controlled by a BehaviourNet Controller BNC MKII operating system (Campden Instruments, Loughborough, UK) were used in this study. Each operant box constituted a sound attenuation chamber that enclosed the 9-hole box made of aluminium on all sides with a clear Perspex lid. The rear wall of each chamber was curved and contained a horizontal array of nine holes (11 mm in diameter, placed 2 mm apart and 15 mm above floor level). Each hole contained photocell infrared beams localised at the front to detect nose pokes. At the rear of each hole a white LED acted as the target visual stimulus. A peristaltic pump delivered liquid reinforcement in the form of strawberry milk (Yazoo^®^, Campina Ltd, Horsham, UK) to a reward magazine at the front of the box, located opposite the 9-hole array. Reward delivery to the magazine was signalled by a light located above the magazine and nose entry into the magazine was detected by an infrared beam located across the opening of the magazine. ‘House lights’ were also located on the side walls of the operant chamber, which illuminated to signal the end of a trial or time out intervals (TOI) within trials. Background noises were provided by an extractor fan and a computer operating system.

#### 2.2.2 Modified Skinner-like operant box configuration

The sixteen 9-hole operant boxes described above were modified to include a Skinner-like configuration with two response locations (photocell holes for recording nose-pokes rather than retractable levers as used in rat apparatus (Dunnett, 1985) were located on the wall directly opposite the side that housed the 9-hole array, one on either side of the reward hopper, as shown in Fig. 1). The two nose poke holes measured 11 mm in diameter and were located 20 mm laterally on either side of the reward magazine and 15 mm above floor level. Both holes contained photocell infrared beams localised at the front to detect nose pokes and a white LED at the back of the hole to act as the target visual stimulus.

### 2.3 Delayed matching and non-matching to position (DMTP and DNMTP) tasks

**Fig. 2:**
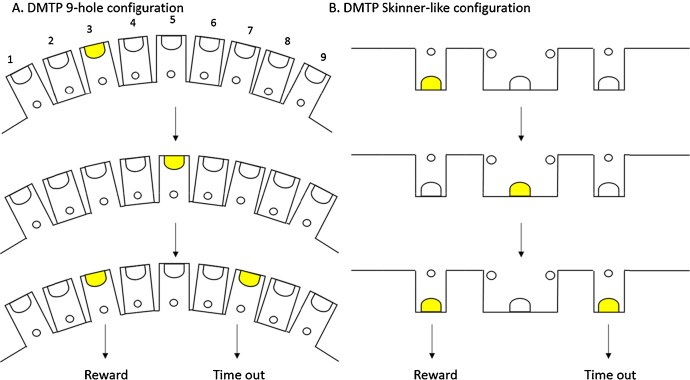
Schematic comparison of the differences in responding required for the delayed matching to position (DMTP) task in different operant apparatus configurations. (A) The responses required in the 9-hole operant configuration are shown, animals are required to poke into a randomly generated hole which can be either hole 3 or hole 7, before responding into hole 5 before being presented simultaneously with both holes 3 and 7. In order to receive a reward the animal must match the response to the stimulus light which was initially presented (in this case hole 3), if an incorrect response is made a 10 s time out is initiated. (B) The responses required in the Skinner-like operant configuration are shown. The animal is required to poke into a randomly generated stimulus light located either to the left or right of the reward magazine. The animal is required to poke back into the magazine to be re-centralised before being presented simultaneously with stimulus lights to both the left and right of the magazine. In order to obtain reward the final response must match the response originally made (in this case to the left of the magazine), if an incorrect response is made then a 10 s time out is initiated.

The delayed matching to position (DMTP) and delayed non-matching to position (DNMTP) tasks were implemented in the same way in each of the different operant configurations. The design of the tasks is such that the mouse is first required to make a response at a specific spatial location, and then must make a subsequent choice between two locations, one of which matches the first forced choice. The mouse must recall the preceding forced response in order to make a correct choice to receive reward: responding to the same response location again in the ‘matching’ task, or to the opposite response location in the ‘non-matching’ task. Delays were introduced between the initial response and the presentation of the choice phase to probe working memory. For both tasks, pairs of forced and choice trials were repeated for a total daily session duration of 30 min. Although the matching and non-matching rules and trial timings were identical, the location of the response holes differed based on the operant configuration used (Fig. 2).

In the conventional 9-hole configuration, a trial was initiated by the illumination of the central hole (hole 5 from left to right), in the 9-hole array. A poke into the central hole led to the illumination of a light, adjacent to the central hole (hole 3 or hole 7) that was randomly selected by the computer. The animal was required to respond into this illuminated hole, and then respond into the central hole again. Once recentralised the animal was presented with the simultaneous illumination of both lights (hole 3 and hole 7) in the choice phase. In the DMTP task the animal was required to match the final response to the same position that it was originally required to poke into, whereas in the DNMTP task a response into the opposite hole was required. If the animal responded correctly it was rewarded with 50 μl of strawberry milk delivered into the magazine. However, if the animal responded incorrectly a 10 s time out was initiated by the illumination of the house light before another trial could be initiated.

The responses required for the DMTP and DNMTP tasks in the Skinner-like configuration of the apparatus were the same as those required in the 9-hole apparatus, however the locations of the required responses differed (Fig. 2). A trial was initiated by the illumination of the magazine. The animal was required to poke into the magazine which led to the random illumination of either the hole to the left or right of the magazine. A nose poke into this hole resulted in the illumination of the magazine, to re-centre the animal, prior to being presented with the simultaneous illumination of both lights (in the choice phase) to the left and right of the magazine. In the DMTP version of the task the animal was required to match the initial response by poking to the same position, and in the DNMTP version of the task the animal was required to respond into the opposite hole into which it originally responded into. If the animal responded correctly it was rewarded with 50 μl of strawberry milk, delivered into the magazine. However, if the animal responded incorrectly a 10 s time out was initiated by the illumination of the house light before another trial could be initiated.

#### 2.3.1 Training

##### 2.3.1.1 Operant apparatus training: Conventional 9-hole configuration

For the first day of training animals were placed into the operant boxes, with non-contingent delivery of 150 μl of strawberry milk into the magazine, which lasted for 20 min. For 7 subsequent training days animals were trained on a single fixed ratio schedule (one poke for one reward) which lasted for 20 min. The central hole of the array was illuminated and the animal was required to poke into the illuminated hole, which resulted in the simultaneous extinguishing of the light and the delivery of 50 μl of reward into the magazine. Animals were encouraged to poke by painting the central hole with strawberry milk. Animals were then trained on a two response schedule for 5 days. A response into the central hole was required, followed by a second response into the randomly selected hole either left or right of the central hole. The location of the second hole was randomly generated via the computer programme. Once the animal had successfully responded twice, it was rewarded with 50 μl of strawberry milk reward, delivered into the magazine. Animals were trained on this 30 min program for 5 days. The final stage of training required three responses to obtain a reward. As before, animals were required to respond into the central hole, followed by a randomly chosen hole either left or right of the central hole and then respond back into the central hole to obtain a 50 μl reward, which was delivered into the magazine. This session lasted for 30 min. Animals were trained on the final phase of the training schedule until they had made over 40 responses on 7 consecutive days.

##### 2.3.1.2 Operant apparatus training: Skinner-like configuration

As with the training for the 9-hole operant apparatus described in Section 2.3.1.1, for the first day of training animals were placed into the operant boxes and non-contingent delivery of 150 μl of strawberry milk into the magazine, occurred for 20 min. For 5 subsequent training days animals were required to nose poke into a randomly chosen light which was illuminated either to the left or right of the magazine, this training programme was 30 min in length.

#### 2.3.2 Testing

Animals were tested on the DMTP and DNMTP tasks described above without delays until an 80% accuracy criteria had been achieved. Upon reaching this criterion, delays were introduced into the DMTP operant task testing schedule. For the C57BL/6J optimisation studies three delay lengths of 0 s, 2 s and 10 s were used. In the Hdh^Q111^ testing four delay lengths of 0, 2, 5 and 10 s were used. The delay lengths were pseudo-randomly implemented by the computer. Hdh^Q111^ mice were tested in 10 day blocks of trials, reversing the choice decision rule between DMTP and DNMTP between consecutive blocks. Thus, mice were initially tested on the DMTP programme for 10 days, then delays were introduced into the testing schedule for an additional 10 days. Then the task was reversed to the DNMTP task initially without delays for 30 days to due difficulty in obtaining 80% accuracy on the reversal task, before delays were introduced for a further 10 days. Then, the task was reversed back to the DMTP testing schedule as described above for 30 days without delays and 10 days with delays. Finally the animals were returned again to the DNMTP rule over the same durations, to allow four complete blocks of testing of acquisition and reversal of the DMTP and DNMTP tasks to be performed in Hdh^Q111^ animals.

### 2.4 Statistical analysis

Statistical analyses were conducted in IBM SPSS Statistics 20 Software. Two or three way analyses of variance (ANOVA) were conducted with daily test session, task reversals, and choice delay interval as repeated measures and genotype and apparatus configurations as between-subjects factors. Where significance was found post-hoc tests with Bonferroni corrections were applied to identify the locus of effects and their interaction(s).

## 3 Results

### 3.1 Comparison of differing operant configurations for use in the delayed matching and non-matching to position tasks in C57BL/6J mice

For comparison between the conventional 9-hole and Skinner-like configurations of the operant apparatus, response accuracy was used as the principle variable used to determine which was the more effective for learning. The number of trials started was used to provide a supplementary measure of task compliance and motivation within the different configurations. The sensitivity of the two configurations to detect working memory effects was measured upon the introduction of delays into the testing schedule. These measures were assessed for both the DMTP and DNMTP tasks.

#### 3.1.1 DMTP acquisition in the 9-hole and Skinner-like operant box configurations

**Fig. 3:**
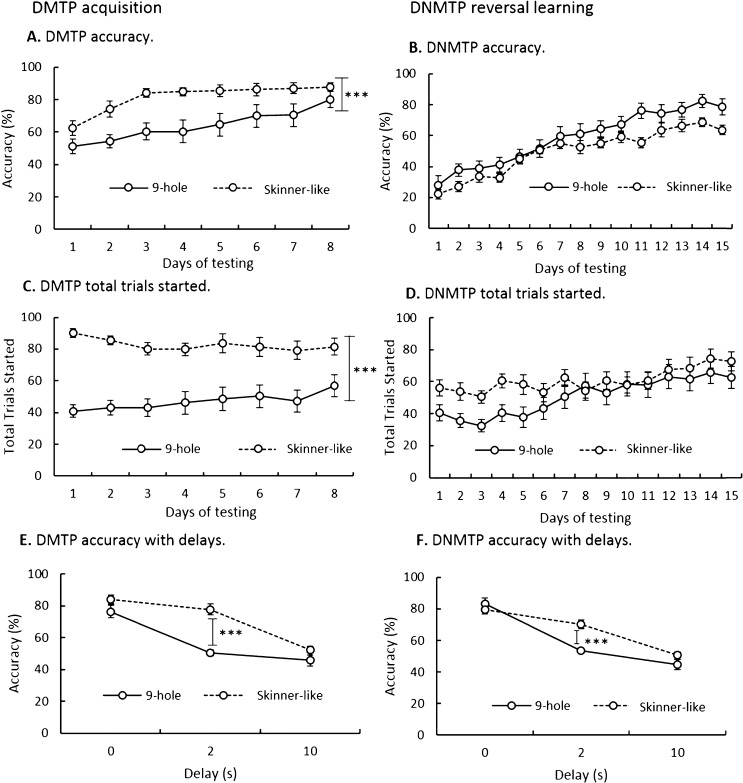
A comparison of delayed matching to position (DMTP) (A, C and E) and non-matching to position (DNMTP) tasks (B, D and F) in the 9-hole operant apparatus and Skinner-like operant apparatuses. (A) DMTP total trials started indicated significantly more trials were started in the Skinner operant apparatus. (B) DNMTP total trials started indicated no significant differences between operant apparatus. (C) DMTP responding accuracy indicated animals were able to reach a higher level of responding in the Skinner operant apparatus. (D) DNMTP responding accuracy indicated no significant differences between operant apparatus. (E) DMTP responding accuracy with delays indicated animals were able to reach a higher level of responding in the Skinner operant apparatus, specifically at the 2 s delay length. (F) DMTP responding accuracy with delays indicated animals were able to reach a higher level of responding in the Skinner operant apparatus, specifically at the 2 s delay length. Error bars indicate ± standard error of the mean. ^***^p < 0.001.

All C57BL/6J mice learned to acquire the DMTP task in both apparatus configurations, performing at close to chance (50%) levels at the start of training and approaching 70–80% levels of accuracy after 8 days of training. Nevertheless, the mice trained in the Skinner-like configuration performed at higher levels overall, exhibiting more rapid learning, higher levels of accuracy overall, and completing more trials per session, in comparison to those trained in the conventional 9-hole configuration (Fig. 3A, Accuracy; *F*_1,27_ = 16.93, *p* < 0.001; Fig. 3C, Trials initiated; *F*_1,27_ = 34.69, *p* < 0.001).

Upon the introduction of delays into the DMTP testing schedule a significant interaction between delay length and apparatus configuration was found (Fig. 3E, Delay × Apparatus; *F*_2,54_ = 8.64, *p* < 0.001). Post-hoc analysis revealed significantly greater response accuracy for the Skinner-like configuration than in the 9-hole configuration specifically at the 2 s delay length (*p* < 0.001), whereas all mice were approaching chance levels of performance (close to 50%) at the longer 10 s delay length.

#### 3.1.2 DNMTP reversal learning in the 9-hole and Skinner-like operant apparatus configurations

Upon reversal of the DMTP task rule to DNMTP all animals initially performed well below chance, perseverating on the old rule with a 20–30% level of accuracy, but then progressively improved performance over 10 days of training and were able to learn the new rule to 70–80% level of accuracy over 15 days of training (Fig. 3B). Although it appeared to be a somewhat lower level of performance asymptote in the Skinner-like configuration, perhaps reflecting the heightened learning of the preceding DMTP task, this difference failed to achieve significance (Fig. 3B, Accuracy; *F*_1,27_ = 4.13, *p* = 0.052), nor were there any differences in the total number of trials started (Trials; Fig. 3D, *F*_1,27_ = 2.00, *p* = n.s.), which was seen in the DMTP version of the task (Fig. 3C, Trials initiated; *F*_1,27_ = 34.69, *p* < 0.001). Nevertheless, upon the introduction of delays, the Skinner-like configuration revealed a significantly higher level of performance at the intermediate 2 s delay (Delay; Fig. 3F, *F*_2,54_ = 11.74, *p* < 0.001).

The data presented (Fig. 3) in this comparison study of operant apparatus configurations, therefore suggests that the results obtained from the DMTP and DNMTP tasks differ depending on the configuration of the operant apparatus used. Specifically animals learn the DMTP task to a higher level of response accuracy in a comparatively shorter time when the Skinner-like configuration of operant apparatus was used. Upon introduction of delays into both the DMTP and DNMTP tasks, significant differences in response accuracy suggest that a higher level of performance can be achieved using the Skinner-like apparatus configuration. Thus, although it is equally feasible to use the DMTP and DNMTP tasks in both the conventional 9-hole and Skinner-like configurations of operant apparatus, animals learned the DMTP task more rapidly and efficiently in the Skinner-like configuration. Therefore, for practical purposes, the Skinner-like configuration of the apparatus is the more efficient to yield robust results and consequently is the version we have selected for testing the DMTP and DNMTP tasks in genetically modified animals of HD.

### 3.2 Delayed matching and non-matching to position in Hdh^Q111^ animals

To determine the suitability of the DMTP and DNMTP tasks for use in examining behavioural deficits in the Hdh^Q111^ mouse model of HD, performance was measured for acquisition of the DMTP task and three subsequent reversal phases of the DMTP and DNMTP tasks. Response accuracy was used to determine learning of the tasks, the total number of trials initiated was used to determine task compliance and the introduction of delays into the testing schedules was used to determine working memory.

#### 3.2.1 Acquisition of the DMTP task reveals significant deficits in learning the DMTP task in the Hdh^Q111/+^ mouse model of HD

**Fig. 4:**
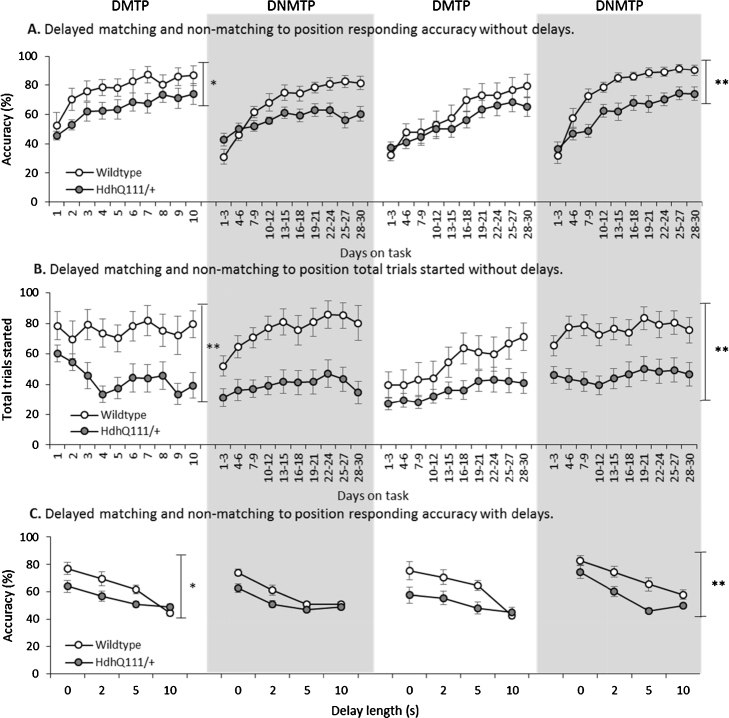
Delayed matching to position (DMTP) and non-matching to position (DNMTP) results for Hdh^Q111^ animals. (A) DMTP and DNMTP responding accuracy without delays. Hdh^Q111/+^ animals were significantly less accurate in the acquisition phase of the DMTP task. During the three reversal learning tasks Hdh^Q111/+^ animals performed significantly less accurately across all tasks than wildtype animals. (B) DMTP and DNMTP total trials started without delays. Hdh^Q111/+^ animals initiated significantly less trials in the acquisition phase of the DMTP task in comparison to wildtype animals. During the three reversal learning tasks Hdh^Q111/+^ animals initiated significantly fewer trials than wildtype animals across all three reversal learning tasks. (C) DMTP and DNMTP responding accuracy with delays. Upon the introduction of delays into the DMTP and DNMTP tasks in the acquisition phase, Hdh^Q111/+^ animals were significantly less accurate than wildtype animals. In the three reversal learning tasks, Hdh^Q111/+^ animals performed significantly less accurately across all delay lengths than wildtype animals. For the DMTP and DNMTP tasks *n* = 21 animals (12 Hdh^Q111/+^ and 9 wildtype). Error bars represent ± standard error of the mean. ^*^p < 0.05, ^**^p < 0.01.

Initial acquisition of the DMTP operant task demonstrated that Hdh^Q111/+^ animals had a significant deficit in acquiring the DMTP task as measured by decreased response accuracy and reduced levels of trial initiations (Fig. 4A, Accuracy; *F*_1,19_ = 5.79, *p* < 0.05, and Fig. 4B, Trials; *F*_1,19_ = 9.41, *p* < 0.01). When delays were introduced Hdh^Q111/+^ animals continued to perform less accurately than wildtype controls at all delay lengths (Fig. 4C, Genotype; *F*_1,19_ = 13.53, *p* < 0.01).

#### 3.2.2 The DMTP and DMTP tasks reveal significant reversal learning and working memory deficits in the Hdh^Q111/+^ mouse model of HD

Upon reversal of the initial DMTP task, to DNMTP, then to DMTP and then finally back to DNMTP the Hdh^Q111/+^ animals continued to exhibit marked deficits in response accuracy irrespective of trial conditions (Fig. 4A, Accuracy; *F*_1,19_ = 11.42, *p* < 0.01), suggesting a consistently significant impairment in task acquisition, reversal learning and asymptotic performance in comparison to wildtype animals. Similarly the Hdh^Q111/+^ animals consistently exhibited a reduced rate of trial initiation in the reversal tasks (Fig. 4B, Trials; *F*_1,19_ = 12.43, *p* < 0.01), and unsurprisingly, the introduction of the delays further exacerbated their deficits at all delay lengths in comparison to wildtype animals (Fig. 4C, Delay; *F F*_1,19_ = 12.83, *p* < 0.01).

## 4 Discussion

We have shown that significantly different results are produced when DMTP and DNMTP protocols are performed in different configurations of operant apparatus. Although the DMTP and DNMTP tasks are viable in both conventional 9-hole and Skinner-like configurations, acquisition of the DMTP task is significantly faster in the Skinner-like configuration. Furthermore, the two configurations have comparatively different levels of sensitivity to detect delay-dependent deficits once delays are introduced into the DMTP and DNMTP testing schedules.

The significant differences demonstrated between the two configurations of the operant apparatus, specifically that the Skinner-like configuration yields more robust results than the conventional 9-hole configuration, provide interesting guides to be considered in the design of operant tasks for cognitive testing in mouse models of neurological diseases. The finding that upon the introduction of delays into the DMTP and DNMTP tasks animals are able to reach a higher accuracy in the Skinner-like operant configuration may be explained by the relative distance between the final response location and the reward location. In the Skinner-like configuration of the DMTP and DNMTP tasks, the reward magazine is located 20 mm laterally from the response stimulus light. Whereas, in the conventional 9-hole configuration the reward magazine is located at the opposite side of the operant box, 14 cm away from the 9-hole array where the response stimulus lights are located. Therefore, the delay between the final response and the collection of the associated reward in the 9-hole configuration of the task may weaken the associative strength of the reward and related acquisition of response, resulting in slower learning. This distance also increases the time for each trial to be completed, which may result in fewer trials over a set session period (of 30 min in the present study). Although the 9-hole operant apparatus configuration offers a greater degree of flexibility and variability in the operant tasks which are able to be performed, for the DMTP and DNMTP protocols described here, the Skinner-like configuration is more sensitive for performing these particular response choice and execution rules.

Furthermore, in comparison to a previous study conducted in mice which used a traditional Skinner operant box configuration with retractable levers to complete the DMTP and DNMTP tasks (Estapé and Steckler, 2001), wildtype animals in the study presented here were able to achieve similar levels of accuracy using a Skinner-like configuration of the task which utilised the species-prepotent nose hole pokes to respond. It is not only more economical to modify existing operant boxes in this way to include a Skinner-like configuration, but the nose poke response holes utilised in this study also provide a naturalistic type of response for mice, who may be impaired in their motor function which may affect their ability to physically lever press. In comparison to rat studies which utilised classical Skinner boxes with retractable levels (Dunnett et al., 1988a, Bushnell, 1990, Cole et al., 1993, Carter et al., 1995, Stephens and Cole, 1996, Yamada et al., 2005), similar results are observed in this study in the Skinner-like operant configuration which utilised nose poke holes for mice, with animals responding on the task to between 80% and 90% accuracy without delays. However, the nose poke response holes used in the Skinner like configuration here are less salient response options than retractable levers.

The use of mediating behaviours to gain an advantage in accurately responding in delayed matching tasks has previously been shown in rats (Dunnett, 1985, Chudasama and Muir, 1997) and pigeons (Blough, 1959). In this case animals were not observed during the task and thus we cannot conclude that the use of a mediating behaviour conferred any specific advantage in either of the operant configurations. However, in future iterations of the task mediating behaviours may be overcome by requiring animals to perform a specific behaviour such as nose poking into the reward magazine during the delay to reduce the opportunity for mediation (Dunnett, 1985, Bushnell, 1990).

When the Hdh^Q111^ mouse model of HD was tested in the Skinner-like configuration on the DMTP and DNMTP tasks, significant deficits were seen in the ability of Hdh^Q111/+^ animals to acquire and perform the DMTP task. Furthermore, when the tasks were reversed significant deficits were seen in Hdh^Q111/+^ animals in terms of response accuracy and the number of trials initiated. Upon the introduction of delays into both the DMTP and DNMTP tasks, a clear effect of delay was demonstrated in all manipulations of the tasks. Although, Hdh^Q111/+^ animals were significantly less accurate than wildtype animals at all delay lengths including at the zero delay, thus suggesting a reduced ability to perform the tasks accurately. Nevertheless, we cannot conclude that the Hdh^Q111^ mice exhibit a specific deficit in working memory performance, since they were impaired at all delays including the shortest, at which the memory load is least. Rather the profile of impairments suggests an executive deficit in learning and performing the choice response rule itself, as well potentially of disturbances in response initiation and motivation.

The reduced ability to acquire, perform and initiate trials in the DMTP and DNMTP tasks may suggest an apathetic phenotype or lack of motivation in Hdh^Q111/+^ mice as suggested by the fewer number of trials performed. Behavioural deficits of this type have previously been seen in people with HD (Rosenblatt and Leroi, 2000, Paulsen et al., 2001, Baudic et al., 2005), although additional behavioural tests will need to be conducted to determine the specificity of deficits within this mouse model. Alternatively, it may be that the increased number of trials initiated by the wildtype animals translates to a practice effect on the performance measure. Therefore, among other variables, future iterations of the task should consider using a set number of trials per session rather than a fixed duration to overcome this potential confound.

The DMTP and DNMTP reversal learning results demonstrate that wildtype animals initially perseverate more on the incorrect response from the previous manipulation as signified by their lower initial baselines on the task reversals. This was expected as these animals have clearly learnt the previous manipulation of the task to a greater degree, and formed a stronger association in learning the rule of the task, than the Hdh^Q111/+^ mice. The Hdh^Q111/+^ animals appear to acquire the task less well, but upon reversal of the task responding accuracy was higher than in wildtype animals, this may be due to a general impairment in rule learning of these tasks. Overall the ability of the Hdh^Q111/+^ animals to learn the reversal task was decreased in comparison to wildtype animals. The reversal learning deficits associated with Hdh^Q111/+^ animals in comparison to wildtype animals may be reflective of perseverance and behavioural inflexibility which have been previously described in people with HD (Craufurd et al., 2001, Hamilton et al., 2003, Ho et al., 2003, Thompson et al., 2014). Therefore, it is possible that Hdh^Q111/+^ animals may be able to perform the task to the same level as wildtype animals, although they may take a significantly longer time to do so. Although this suggestion is unlikely as Hdh^Q111/+^ animals seem to reach a plateau in both responding accuracy and the number of trials started over the 30 days of testing on each reversal manipulation. Furthermore, there seems to be a greater deficit in response accuracy when the task is reversed from DMTP to DNMTP, in comparison to when the task is reversed from DNMTP to DMTP. This trend has been previously reported in rat studies (Dunnett et al., 1988b, Dunnett et al., 1989) and may be due to the different strategies used in learning the DMTP and DNMTP rules.In comparison to other tasks of reversal learning in mouse models of HD, such as the Morris water maze and the water T-maze (Lione et al., 1999, Van Raamsdonk et al., 2005, Brooks et al., 2012a, Brooks et al., 2012b, Brooks et al., 2012c, Brooks et al., 2012d); the DMTP and DNMTP operant tasks presented here are comparatively easier conduct and subject animals to less stress and distress than water based tasks. Although dry maze tasks could be utilised to investigate reversal learning in mouse models of HD encouraging the mice to perform the task can be troublesome and behavioural testing of this nature is particularly labour intensive.

The DMTP and DNMTP task protocols presented here therefore provide a highly sensitive, robust and reproducible method to test spatial learning and its reversal and short-term memory function in genetically modified mice. The results demonstrate that this method of behavioural testing would also be suitable to test pharmacological therapeutic interventions in mouse models of HD and is also translatable to mouse models of other neurological diseases.

## 5 Conclusions

The 9-hole operant apparatus has been utilised to test delayed matching and delay non-matching to position (DMTP and DNMTP) protocols in mice in two different configurations: one using the conventional 9-hole stimulus response array, and the other a Skinner-like configuration in which two response holes were located either side of the central reward hopper. Whilst mice were able to learn the DMTP and DNMTP tasks in both configurations, the Skinner-like configuration produced more efficient, rapid, robust and sensitive results in comparison to the conventional 9-hole configuration. Further testing of the DMTP and DNMTP tasks using the latter configuration demonstrated that the Hdh^Q111^ mouse model of HD exhibited significant deficits in the acquisition of the DMTP task and subsequent reversal learning in comparison to their wildtype controls. Our data suggest that the DMTP and DNMTP operant procedures described here can provide valid and robust tests of cognition, executive function and working memory for use in murine models of neurodegenerative diseases, including HD.

## Conflict of interest statement

None to declare.
